# Role of Hypoxia-Inducible Factor-1 Alpha in Endometrial Decidualization

**DOI:** 10.3390/biom16101483

**Published:** 2026-10-13

**Authors:** Defan Wang, Zhenghong Zhang, Renfeng Xu, Zhengchao Wang

**Affiliations:** 1 Fujian Provincial Key Laboratory of Reproductive Health Research, School of Medicine, Xiamen University, Xiamen 361102, China; defanwang@126.com; 2 Provincial Key Laboratory for Developmental Biology and Neurosciences, College of Life Sciences, Fujian Normal University, Fuzhou 350007, China; zhangzh@fjnu.edu.cn; 3 Fujian Key Laboratory of Integrative Medicine on Geriatrics, College of Integrative Medicine, Academy of Integrative Medicine, Fujian University of Traditional Chinese Medicine, Fuzhou 350122, China; renfengxu@fjtcm.edu.cn

**Keywords:** hypoxia-inducible factor 1α, endometrial decidualization, metabolic reprogramming, immune regulation, epigenetic regulation

## Abstract

Endometrial decidualization is a hormonally programmed differentiation and tissue-remodeling process that prepares the endometrium for embryo implantation and supports early pregnancy. It involves coordinated changes in endometrial stromal cells, the extracellular matrix, vascular function, metabolism, and local immune composition. Hypoxia-inducible factor-1α (HIF-1α) is an oxygen-sensitive transcription factor that responds to changes in tissue oxygenation and metabolic state. Evidence from endometrial stromal-cell models and reproductive tissues suggests that HIF-1α may participate in decidual cell differentiation, metabolic adaptation, angiogenic signaling, and immune regulation. However, the strength of evidence differs substantially among these processes, and findings from trophoblasts, placenta, immune cells, or non-reproductive tissues cannot automatically be interpreted as direct evidence for decidual stromal-cell mechanisms. This review summarizes current evidence for HIF-1α in endometrial decidualization, distinguishes direct endometrial findings from mechanistic extrapolation, and considers the interaction of HIF-1α with metabolic, vascular, immune, and epigenetic pathways. Overall, HIF-1α is best considered a context-dependent component of the decidualization network rather than an independent master regulator. Defining its cell-specific and temporal functions, including its relationship with HIF-2α, will be important for determining whether HIF-directed interventions can be developed without disrupting physiological reproductive adaptation.

## 1. Introduction

Decidualization is a pivotal physiological event in human embryo implantation and the establishment of pregnancy. Fundamentally, it is a process of programmed differentiation wherein endometrial stromal cells (ESCs) transform into highly secretory decidual stromal cells (DSCs) in response to stimulation by progesterone and cAMP. Decidualization not only determines the success of embryo implantation but also directly influences placentation, maternal–fetal immune tolerance, and the maintenance of pregnancy. Consequently, it is regarded as a critical window for the success of early pregnancy [1]. Traditional theory holds that decidualization is primarily regulated by ovarian hormones, with progesterone acting via PR signaling axis, and inducing the expression of classic decidualization markers such as PRL and IGFBP1, thereby facilitating the endometrium’s transition from a proliferative state to a secretory, supportive state. However, increasing evidence in recent years suggests that endocrine signaling alone cannot fully account for the complexity of decidualization. Local oxygen metabolism, metabolic reprogramming, and the remodeling of the immune microenvironment also play central regulatory roles during this process [2].

During the menstrual cycle, endometrial oxygen availability changes with cyclical vascular tone and tissue remodeling. Evidence supports a transient physiological hypoxic response around menstruation and endometrial repair, whereas the exact oxygen tension of human menstrual endometrium remains incompletely defined. Measurements in non-menstrual endometrium have reported oxygen tensions around 15~19 mmHg, and experimental work indicates that vasoconstriction and tissue breakdown during menstruation can generate a more hypoxic local environment [3,4,5]. Maybin et al. demonstrated that hypoxic stress regulates vascular endothelial growth factor (VEGF) and stromal cell-derived factor-1/CXCL12 in human endometrial stromal cells, supporting a functional role for hypoxia in endometrial repair [3,4,5,6]. Research indicates that moderate hypoxia significantly enhances the decidualization capacity of ESCs, whereas exposure to abnormally high oxygen levels inhibits embryo implantation and endometrial receptivity, suggesting that hypoxia is not merely a passive consequence but a crucial regulatory factor actively involved in initiating decidualization [7,8]. During this process, hypoxia-inducible factor (HIF)-1α is rapidly stabilized and activated. By transcriptionally regulating genes associated with glucose metabolism, angiogenesis, cell survival, and inflammatory responses, it acts as a central hub linking oxygen sensing to tissue remodeling [9].

HIF-1α is a classic oxygen-sensitive transcription factor. Under normoxic conditions, HIF-1α undergoes hydroxylation by PHDs, followed by VHL-mediated ubiquitination and degradation. Under hypoxic conditions, PHD activity decreases, allowing HIF-1α to stabilize, accumulate, and translocate into the nucleus. It forms a heterodimer with HIF-1β and binds to HREs to initiate the transcription of numerous target genes [10,11]. Recent studies have demonstrated that HIF-1α exhibits highly dynamic expression patterns within the female reproductive system, playing a pivotal role in processes such as menstrual repair, embryo implantation, decidualization, and placentation. Specifically within the endometrium, HIF-1α not only regulates VEGF-mediated angiogenesis but also influences endometrial receptivity and decidual differentiation by modulating key molecules such as HOXA10, IGFBP1, and PKM2 [12]. A study on recurrent implantation failure (RIF) found that HIF-1α expression in the endometrium of RIF patients was significantly reduced, accompanied by a concomitant decrease in glycolysis-related genes such as PKM2, LDHA, and HOXA10, suggesting that HIF-1α may participate in decidualization and embryo implantation by regulating metabolic reprogramming [13].

As research has deepened, it has become increasingly clear that decidualization is a dynamic process characterized by high energy consumption and systemic remodeling. During the differentiation of ESCs into DSCs, the cells shift from relying primarily on oxidative phosphorylation to a state dependent on glycolysis, accompanied by increased lipid and glutamine metabolism as well as elevated lactate production. This metabolic reprogramming not only meets the energy demands of rapid differentiation but also regulates immune cell recruitment and epigenetic modifications through metabolic intermediates [14]. As a central regulator of metabolism, HIF-1α induces the expression of glycolytic enzymes such as GLUT1, HK2, LDHA, and PKM2, and promotes an aerobic glycolysis like metabolic pattern, driving the expression of decidualization-related genes and cellular phenotypic transitions [15]. Furthermore, lactate accumulation can induce immune tolerance by acidifying the microenvironment and stabilizing the functions of Tregs and dNK cells, further reinforcing maternal–fetal immune homeostasis [16,17].

Beyond its metabolic roles, HIF-1α is deeply involved in the immune remodeling associated with decidualization. The establishment of a normal pregnancy requires the formation of a highly tolerant maternal–fetal interface. Notably, immune cells including dNK cells, macrophages, dendritic cells and Tregs, account for approximately 40–50% of the cells within decidual tissue [18,19,20]. Research indicates that HIF-1α promotes the polarization of immune cells toward a tolerogenic phenotype by regulating the expression of chemokines, inflammatory cytokines, and immune checkpoint molecules. For instance, HIF-1α can induce the transition of macrophages to the M2 phenotype and enhance the secretion of immunosuppressive and angiogenic factors such as IL-10 and VEGF, maintaining a low-inflammation state in decidual tissue [21,22,23,24,25]. Concurrently, HIF-1α forms a regulatory network involving autophagy, NLRP3 inflammasome and ROS signaling, contributing to the maintenance of immune homeostasis at the maternal–fetal interface [26,27]. These findings suggest that decidualization is not merely a cellular differentiation event but a complex systemic process involving the coordinated remodeling of metabolism, inflammation and immune tolerance.

In recent years, advancements in multi-omics and single-cell sequencing technologies have further deepened our understanding of the role of HIF-1α in early pregnancy. Recent studies suggest that HIF-1α and HIF-2α perform non-redundant functions across temporal and spatial dimensions. Specifically, HIF-1α primarily regulates early decidualization, metabolic adaptation and immune tolerance, whereas HIF-2α is involved in later-stage trophoblast invasion and the formation of placental tissue architecture [28]. This perspective indicates that HIF signaling is not merely a simple hypoxic response system but a central regulatory axis coordinating the multifaceted biological processes of early pregnancy. Consequently, redefining decidualization as a HIF-1α-driven system-level tissue remodeling process helps transcend traditional endocrine-centric theoretical frameworks, offering a new theoretical basis and potential therapeutic targets for pregnancy-related conditions such as RIF, recurrent pregnancy loss, and preeclampsia [29,30,31].

## 2. Literature Search and Scope

This review was developed from a focused search of PubMed and related biomedical literature using combinations of terms including “HIF-1α”, “hypoxia”, “endometrial decidualization”, “endometrial stromal cells”, “decidual stromal cells”, “metabolism”, “glycolysis”, “lactate”, “immune regulation”, “epigenetic regulation”, “implantation”, and “pregnancy-related disorders”. Recent literature was prioritized, while foundational studies were retained when necessary to define established mechanisms.

Evidence directly obtained from human endometrial stromal/decidual cells was prioritized for conclusions concerning decidualization. Studies from trophoblasts, placenta, immune cells, vascular tissues, or other organs were used primarily to discuss mechanistic plausibility and are identified as indirect evidence where appropriate. This article is a focused narrative review rather than a systematic review or meta-analysis; no formal risk-of-bias assessment or quantitative evidence synthesis was performed.

## 3. Physiological Sequence and Tissue Remodeling During Endometrial Decidualization

Decidualization is a temporally ordered differentiation program that begins during the mid-to-late secretory phase and continues through the peri-implantation period. Progesterone and cyclic AMP (cAMP) signaling initiate ESC differentiation, accompanied by cell-cycle exit, cytoskeletal reorganization, enlargement of the stromal-cell compartment, and acquisition of a secretory phenotype characterized by increased prolactin (PRL) and insulin-like growth factor-binding protein 1 (IGFBP1). These molecular changes occur progressively rather than as a single switch and are coordinated with extracellular-matrix remodeling and altered paracrine signaling.

At the tissue and cellular levels, decidualization involves coordinated remodeling of stromal cells, epithelial cells, endothelial and perivascular cells, and resident immune populations. Stromal cells acquire decidual morphology and secretory function; the extracellular matrix is reorganized; vascular density and endothelial function adapt to changing tissue demands; and uterine natural killer (uNK/dNK) cells, macrophages, and other immune populations undergo phenotypic and functional changes that support tissue homeostasis and maternal–fetal immune adaptation. These processes are temporally coupled but are not necessarily controlled by a single upstream pathway.

Decidualization is also accompanied by substantial metabolic and vascular adaptation. Glucose utilization, lipid handling, mitochondrial activity, amino-acid metabolism, and lactate production are remodeled to meet the energetic and biosynthetic requirements of differentiating stromal cells. In parallel, endometrial perfusion and oxygenation fluctuate across the menstrual cycle and peri-implantation period. Current human evidence supports transient hypoxia-related signaling, particularly during menstruation, but direct quantitative measurements across the entire cycle remain limited. Therefore, a fixed oxygen tension should not be assumed for every stage of decidualization. This temporal and multicellular framework provides the appropriate context for evaluating the contribution of HIF-1α.

## 4. Expression Characteristics and Activation Mechanisms of HIF-1α

**Figure 1:**
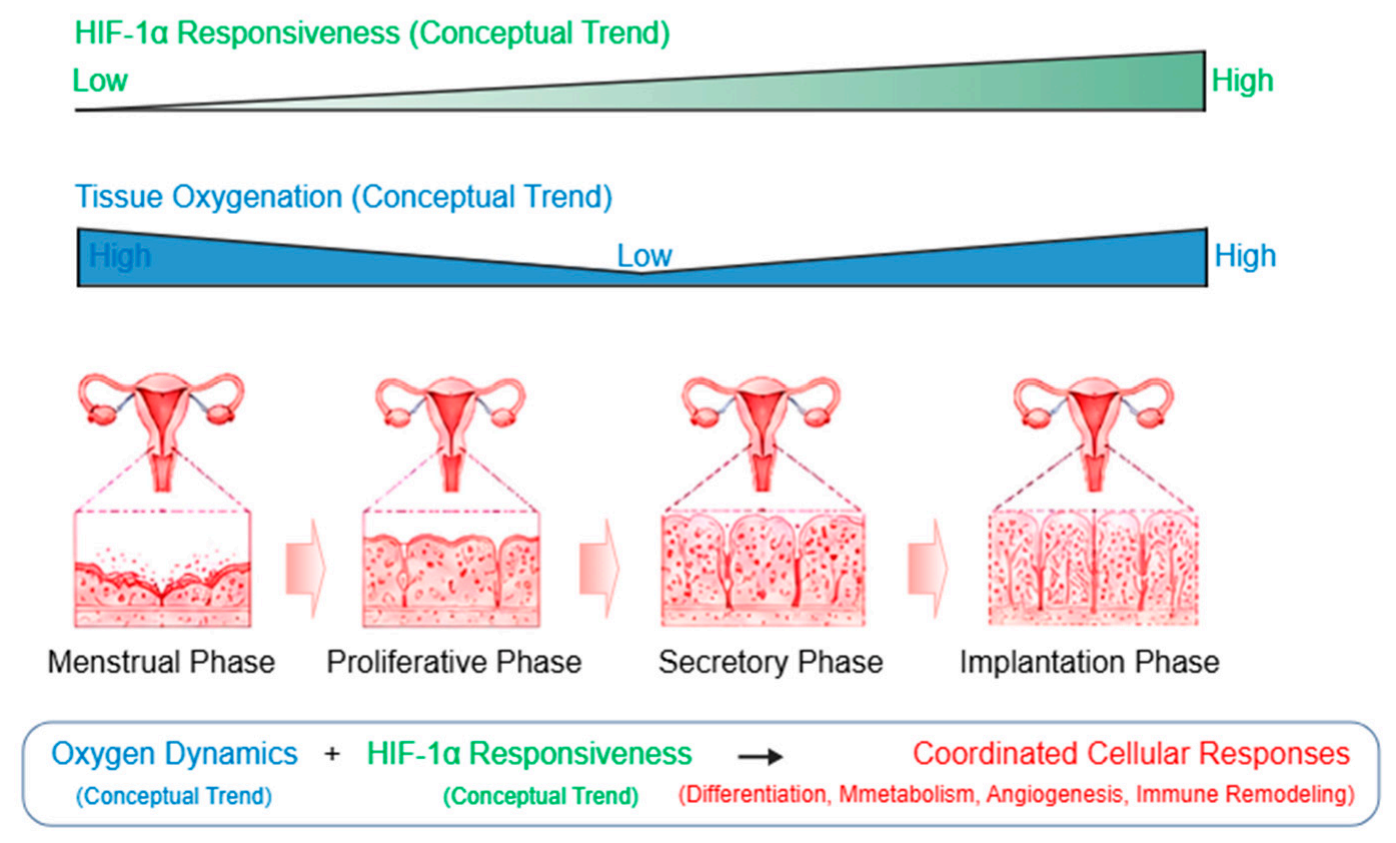
HIF-1α and oxygen dynamics across the menstrual cycle. The schematic depicts conceptual changes in tissue oxygenation and HIF-1α responsiveness across the menstrual and implantation phases. It also identifies major cell populations in which HIF signaling may be relevant, including endometrial stromal/decidual cells, endothelial cells, and decidual immune cells. The oxygen trajectory is presented as a conceptual physiological trend rather than a quantitative measurement. Created with BioGDP.com (accessed on 7 August 2026).

HIF-1α is a pivotal oxygen-responsive transcription factor whose abundance and activity vary with tissue oxygenation, endocrine signals, and cellular metabolic state. In the human endometrium, HIF-1α-associated hypoxic responses show menstrual-cycle-dependent changes, with hypoxia-regulated factors particularly evident around menstruation and endometrial repair (Figure 1) [3,4,5,6]. Available evidence also indicates HIF-1α expression or activity in stromal and glandular epithelial compartments and in vascular-associated regions, although cell-specific expression and functional requirements are not equally established for every menstrual phase. These observations support a dynamic role for HIF-1α in the endometrium, but they do not by themselves establish HIF-1α as the dominant regulator of decidualization.

**Figure 2:**
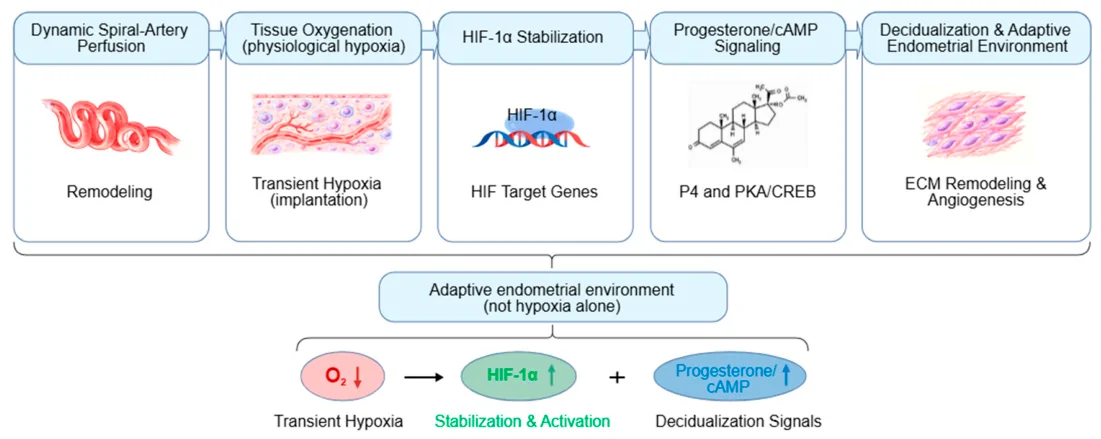
Physiological hypoxia around implantation. Dynamic spiral-artery perfusion and tissue oxygenation can create a transient hypoxic stimulus that favors HIF-1α stabilization. This signal is integrated with progesterone/cAMP-driven decidualization and contributes to the adaptive endometrial environment. The figure does not imply that hypoxia alone is sufficient to induce decidualization. Created with BioGDP.com (accessed on 7 August 2026).

Around the peri-implantation period, the endometrium undergoes coordinated vascular, stromal, epithelial, and immune remodeling. Physiological hypoxic signaling can stabilize HIF-1α and regulate genes involved in angiogenesis, metabolism, and tissue repair. However, the exact oxygen tension of the human endometrium during this interval has not been established as a fixed 2–8% value [3,4,5,6]. Hypoxic stress in human endometrial stromal cells can increase HIF-1α-dependent VEGF expression, providing a mechanistic link between oxygen sensing and local vascular responses [3,4,5]. These findings support a role for HIF-1α-associated signaling in endometrial adaptation while avoiding the assumption that all peri-implantation remodeling is directly HIF-1α-dependent (Figure 2) [32,33,34,35].

**Figure 3:**
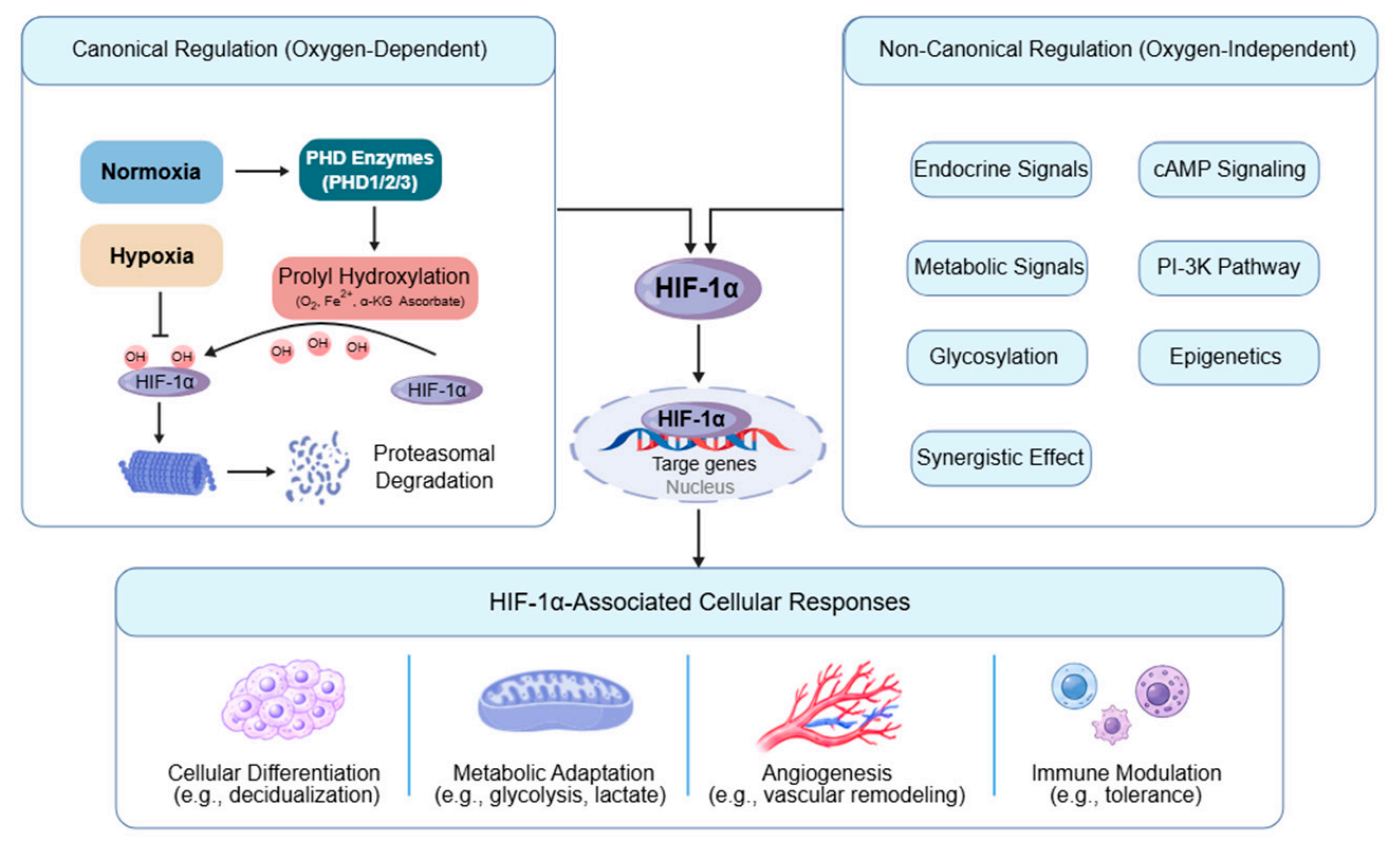
Canonical and non-canonical regulation of HIF-1α. Canonical regulation involves oxygen-dependent PHD hydroxylation and VHL-mediated degradation, whereas non-canonical inputs include PI3K/AKT/mTOR, cAMP/PKA, MAPK, and metabolic signals. The simplified diagram emphasizes pathway architecture; detailed mechanisms are provided in the main text. Created with BioGDP.com (accessed on 7 August 2026).

The stability of HIF-1α is primarily regulated by prolyl hydroxylase domain proteins (PHDs) and the von Hippel–Lindau (VHL) ubiquitination system (Figure 3) [36,37,38,39,40,41,42,43,44]. Under normoxic conditions, PHDs hydroxylate HIF-1α, facilitating VHL-mediated ubiquitination and proteasomal degradation. Under hypoxic conditions, reduced PHD activity permits HIF-1α stabilization and nuclear accumulation.

Regarding HIF-1α and HIF-2α, they have overlapping but distinct functions at the maternal–fetal interface. HIF-1α and HIF-2α are closely related oxygen-sensitive transcription factors that share HIF-β (ARNT)-dependent transcriptional machinery but are not functionally interchangeable. Current reproductive evidence indicates that HIF-1α is broadly associated with acute or inducible hypoxic responses, whereas HIF-2α may show more sustained, cell-selective activity in vascular and trophoblast compartments. At the maternal–fetal interface, both isoforms can regulate overlapping hypoxia-responsive programs, but their relative contribution depends on cell identity, oxygen tension, duration of hypoxia, developmental stage, and transcriptional cofactors [28]. Therefore, observations of HIF-2α activity in trophoblasts or placental vascular cells should not be assumed to represent the same mechanism operating in decidualizing endometrial stromal cells. Direct comparative studies in decidual stromal cells remain limited. An important priority is to define the temporal sequence and cell-specific functions of HIF-1α versus HIF-2α during decidualization and early pregnancy.

**Figure 4:**
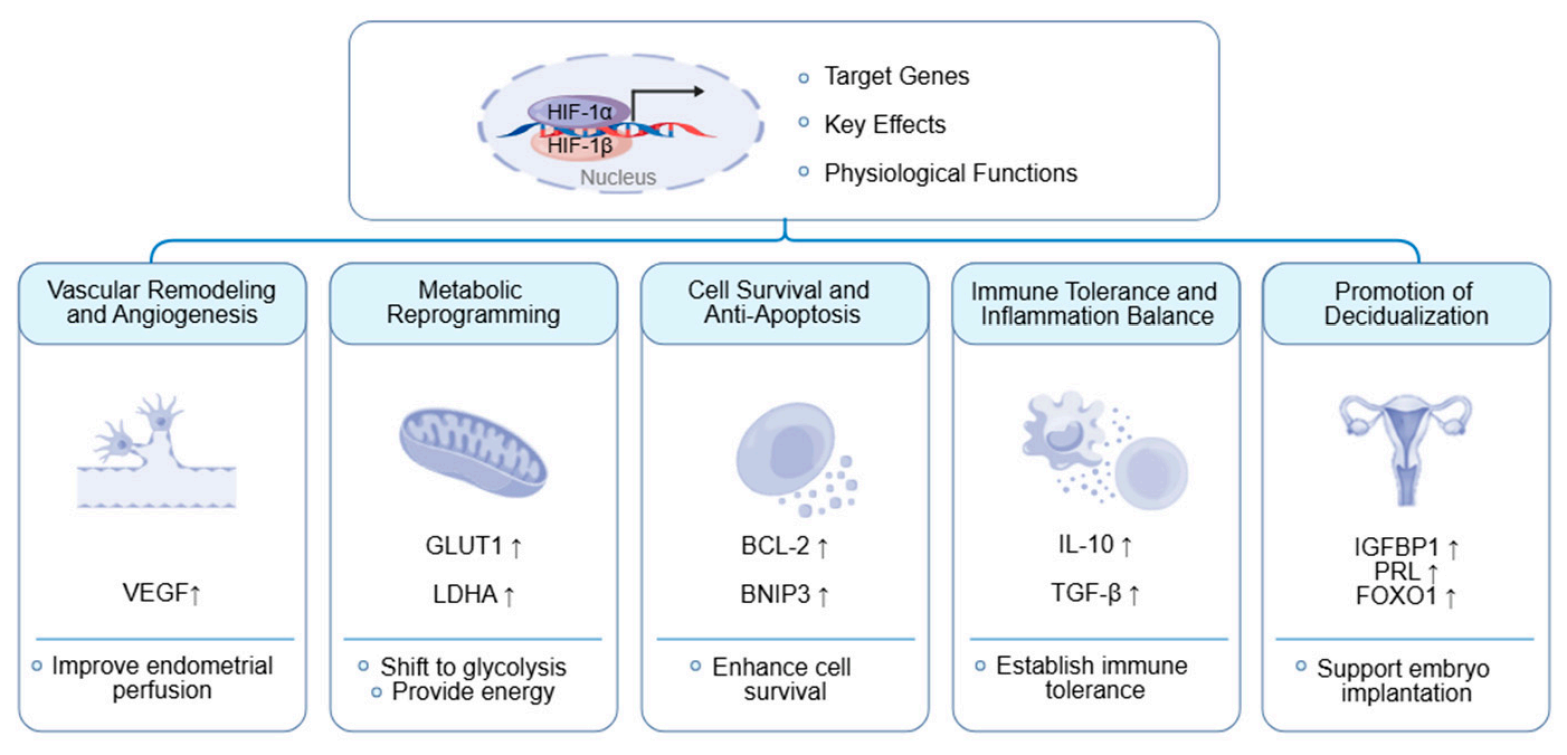
HIF-1α-associated target processes during decidualization. HIF-1α may influence decidual differentiation, metabolic adaptation, cell survival, vascular signaling, and immune regulation. The figure summarizes functional domains rather than establishing that every downstream process is directly HIF-1α-dependent in human decidual stromal cells. Created with BioGDP.com (accessed on 7 August 2026).

Overall, current evidence indicates that HIF-1α activity in the endometrium is dynamically regulated by oxygen availability together with endocrine, metabolic, and local signaling inputs (Figure 4) [3,4,5]. Rather than representing a fixed hierarchical pathway, these regulatory interactions are likely to vary across menstrual phase and cell type. Accordingly, HIF-1α should be viewed as one component of a broader endometrial regulatory network, with the strength of evidence differing among stromal, epithelial, vascular, and immune compartments.

## 5. HIF-1α-Associated Processes During Endometrial Decidualization

**Figure 5:**
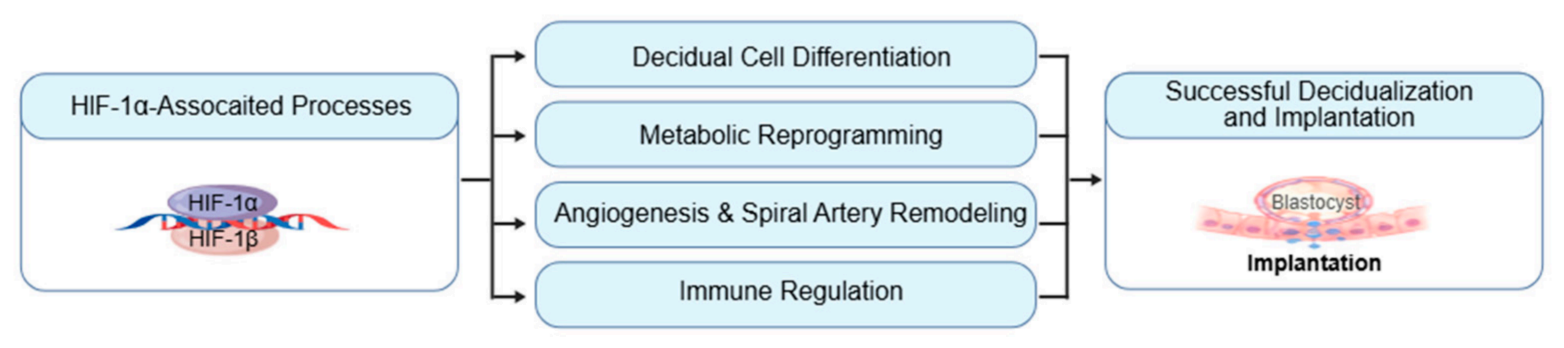
Context-dependent role of HIF-1α in decidualization. HIF-1α is positioned as an integrating node connecting decidual differentiation, metabolic adaptation, vascular/tissue responses, and maternal–fetal immune regulation. The final decidual phenotype reflects the combined effects of multiple pathways rather than a single HIF-1α-driven cascade. Created with BioGDP.com (accessed on 7 August 2026).

Endometrial decidualization is a tissue remodeling process coordinately regulated by hypoxia, metabolism, and immune mechanisms. HIF-1α serves as a central node in this complex network [2], participating not only in angiogenesis but also in key processes such as decidual cell differentiation, metabolic reprogramming, tissue remodeling, and the establishment of maternal–fetal immune tolerance (Figure 5) [12].

### 5.1. Contribution to Decidual Cell Differentiation

**Figure 6:**
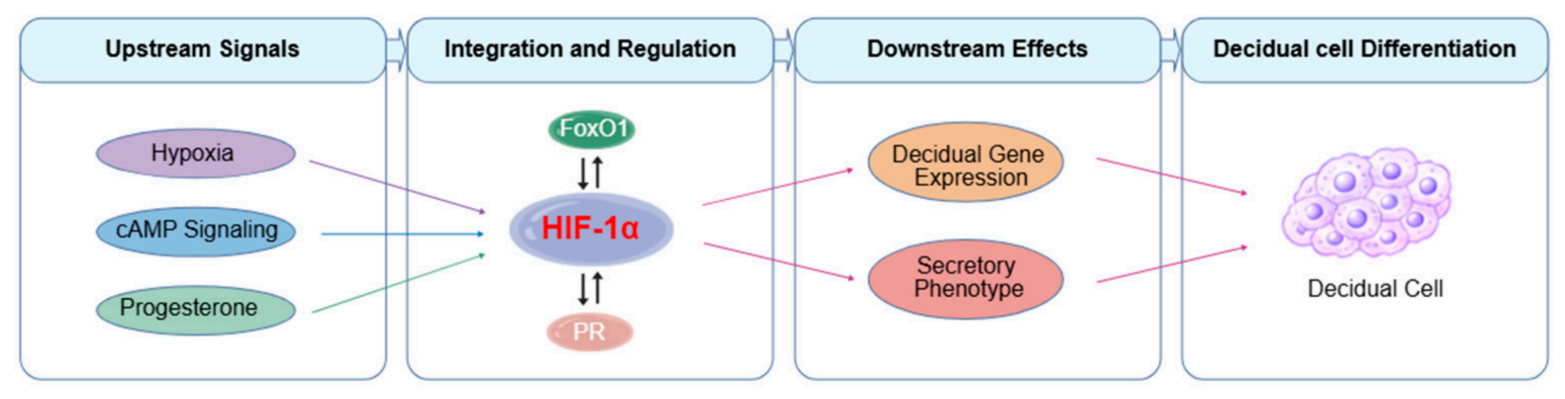
HIF-1α-associated regulation of decidual cell differentiation. Hypoxia, cAMP, and progesterone-related signals can converge on HIF-1α and interact with transcriptional regulators such as FoxO1 and progesterone receptor signaling. Downstream changes include decidual gene expression and the secretory stromal-cell phenotype. Created with BioGDP.com (accessed on 7 August 2026).

Decidualization is fundamentally the process by which ESCs transform into highly secretory DSCs in response to progesterone and cAMP stimulation. HIF-1α has been investigated as a potential modulator of decidualization-associated genes, including PRL and IGFBP1, and may intersect with transcriptional regulators such as FoxO1 (Figure 6) [1]. Chen et al. have reported that hypoxia was accompanied by increased HIF-1α expression and higher PRL and IGFBP1 mRNA levels in ESCs. Inhibitor-based and siRNA experiments have also suggested that HIF-1α contributes to decidualization capacity in the tested models [38,39,40,41,42,43]. These findings support a contributory role, but do not establish that HIF-1α alone determines the differentiation potential of human ESCs across physiological contexts.

FoxO1 is a pivotal transcriptional regulator in the process of decidualization. It participates in cell cycle exit and modulates genes associated with antioxidation, autophagy and cell survival [44,45]. Although HIF-1α and FoxO1 can converge on overlapping adaptive programs, the available evidence does not establish a universal bidirectional HIF-1α-FoxO1 feedback circuit in human decidualizing stromal cells. HIF-1α may nevertheless interact with decidualization-related transcriptional networks involving HOXA10, STAT3, and C/EBPβ [46,47,48,49].

Notably, there is significant synergy between HIF-1α and PR signaling. Karar et al. demonstrates that progesterone not only induces decidualization via PR signaling but also enhances HIF-1α translation efficiency through the PI-3K/AKT/mTOR pathway [50,51,52]. Concurrently, HIF-1α boosts the transcriptional activity of PR target genes, collectively maintaining the differentiated state and functional stability of decidual cells [13]. The PR-HIF-1α cooperative axis is considered a crucial molecular basis for the establishment of endometrial receptivity. Recent studies on patients with RIF have further demonstrated a significant reduction in both HIF-1α and PR expression in the endometrium, accompanied by impaired decidualization capacity [53,54,55], suggesting that HIF-1α dysfunction may be a key mechanism underlying pregnancy failure.

### 5.2. Metabolic Reprogramming During Decidualization: Contribution of HIF-1α

**Figure 7:**
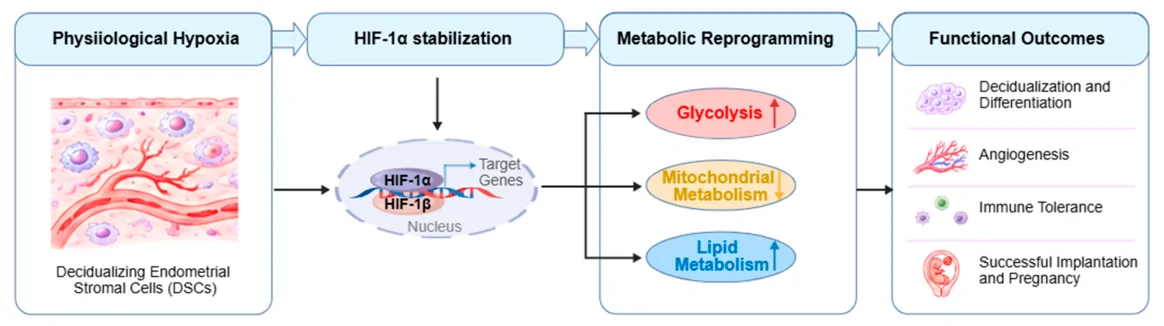
HIF-1α and metabolic adaptation during decidualization. HIF-1α can promote glucose uptake and glycolytic gene expression, including GLUT1, HK2, and LDHA, thereby supporting lactate production and biosynthetic metabolism. The diagram does not imply complete replacement of oxidative phosphorylation by glycolysis. Created with BioGDP.com (accessed on 7 August 2026).

Decidualization is a highly energy-demanding biological process. During the differentiation, ESCs must rapidly synthesize large quantities of proteins, lipids and extracellular matrix components, necessitating significant metabolic reprogramming [14]. HIF-1α plays a central role in regulating this metabolic shift, and by inducing the expression of glycolysis-related enzymes, it drives ESCs to adopt a Warburg-like metabolic state resembling that of tumor cells (Figure 7) [15].

During decidualization, available studies suggest changes in glucose utilization and other metabolic pathways, although the relative contributions of oxidative phosphorylation and glycolysis may vary with model and differentiation stage. HIF-1α can increase the expression of glycolysis-associated genes, including GLUT1, HK2, PFKFB3, and LDHA, and may thereby contribute to glucose uptake and lactate production [6]. In other experimental systems, HIF signaling has been linked to changes in mitochondrial pyruvate oxidation, TCA-cycle activity, and ROS handling [56,57]; the extent to which these effects occur in human decidualizing stromal cells requires further direct validation. Metabolic intermediates may support biosynthesis during differentiation [58,59,60,61]. Lactate has also been reported to influence PHD activity and HIF stability in selected contexts [62,63], but a self-sustaining HIF-1α-glycolysis-lactate loop has not been established in human decidualizing stromal cells. Lactate-associated effects on inflammatory and immune-cell phenotypes have been described in other settings [16,18], but these findings should not be taken as proof that HIF-1α-driven lactate production establishes maternal–fetal immune tolerance in the endometrium.

In addition to glucose metabolism, lipid and glutamine pathways may contribute to the metabolic adaptation accompanying decidualization. Studies in selected cellular systems link HIF-1α to genes involved in fatty-acid uptake and lipid storage [64,65,66], while glutamine metabolism can support glutathione homeostasis and redox buffering [67,68,69,70,71,72,73,74,75]. The extent to which these pathways are directly controlled by HIF-1α in human decidualizing stromal cells remains insufficiently defined. These mechanisms should therefore be regarded as plausible contributors rather than evidence that HIF-1α is the central coordinator of all metabolic adaptation during decidualization.

### 5.3. Angiogenesis and Spiral Artery Remodeling: Distinct but Interacting Processes

#### 5.3.1. HIF-1α and Endometrial Angiogenesis

**Figure 8:**
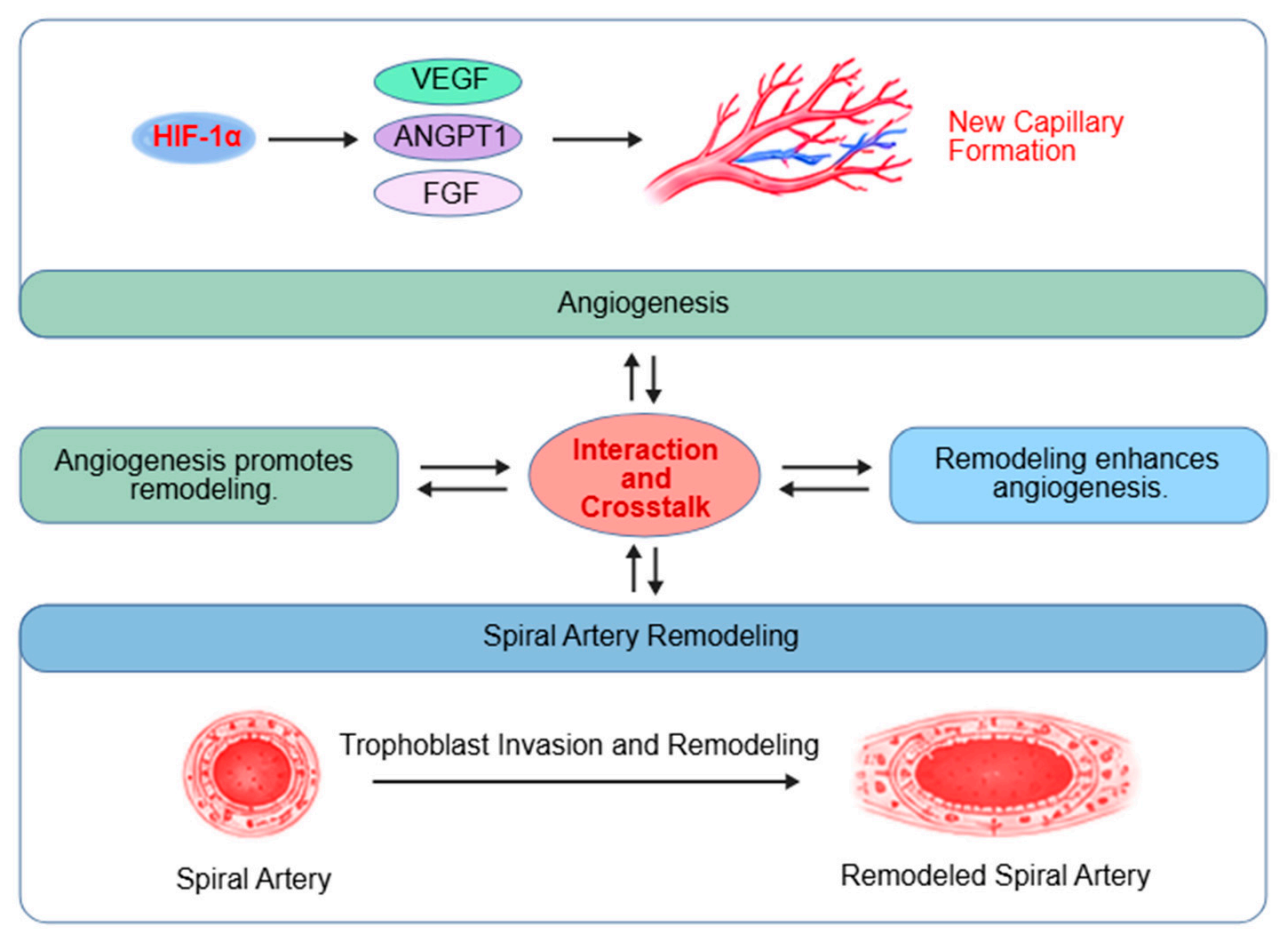
Distinct but interacting processes of angiogenesis and spiral artery remodeling. HIF-1α-associated VEGF/ANGPT signaling can contribute to endometrial microvascular adaptation and angiogenesis. Spiral artery remodeling is a related but mechanistically distinct process involving decidual cells, vascular cells, decidual NK cells, and extravillous/endovascular trophoblasts. Impaired angiogenesis may alter the decidual microenvironment but should not be interpreted as a direct cause of failed spiral artery remodeling. Created with BioGDP.com (accessed on 7 August 2026).

Endometrial angiogenesis refers to the formation, expansion, and functional adaptation of the local microvascular network. HIF-1α can contribute to this process through transcriptional regulation of angiogenic mediators, particularly VEGF and angiopoietin-related pathways. The most direct cellular context is the endothelial compartment, although decidual stromal cells can provide paracrine signals that influence endothelial behavior (Figure 8). The available evidence supports a contributory role rather than a single-cell-type requirement for HIF-1α.

VEGF is a major downstream target of HIF-1α. Under hypoxic conditions, HIF-1α can enhance VEGF expression and thereby support endothelial-cell proliferation, migration, permeability, and microvascular adaptation [3,4,5]. Other HIF-responsive mediators, including angiopoietins, matrix metalloproteinases, and integrin-associated pathways, may further influence the extracellular environment. These effects provide a mechanistic link between HIF-1α activity and endometrial angiogenesis, but they should not be equated with direct control of spiral artery remodeling.

#### 5.3.2. HIF-1α and Spiral Artery Remodeling

Spiral artery remodeling is biologically distinct from angiogenesis and involves coordinated changes in vascular smooth-muscle and endothelial cells, decidual stromal cells, decidual/uNK cells, and, particularly after implantation, extravillous and endovascular trophoblasts. HIF-1α may influence this process indirectly through the decidual microenvironment and directly within some participating cell populations, but current evidence does not support a simple linear model in which impaired angiogenesis causes failed spiral artery remodeling [28,76,77]. Conversely, abnormal oxygen signaling may affect both processes in parallel. The magnitude and direction of HIF-1α effects also depend on cell type, oxygen tension, and developmental stage.

### 5.4. Immune Regulation at the Decidual/Maternal–Fetal Interface

**Figure 9:**
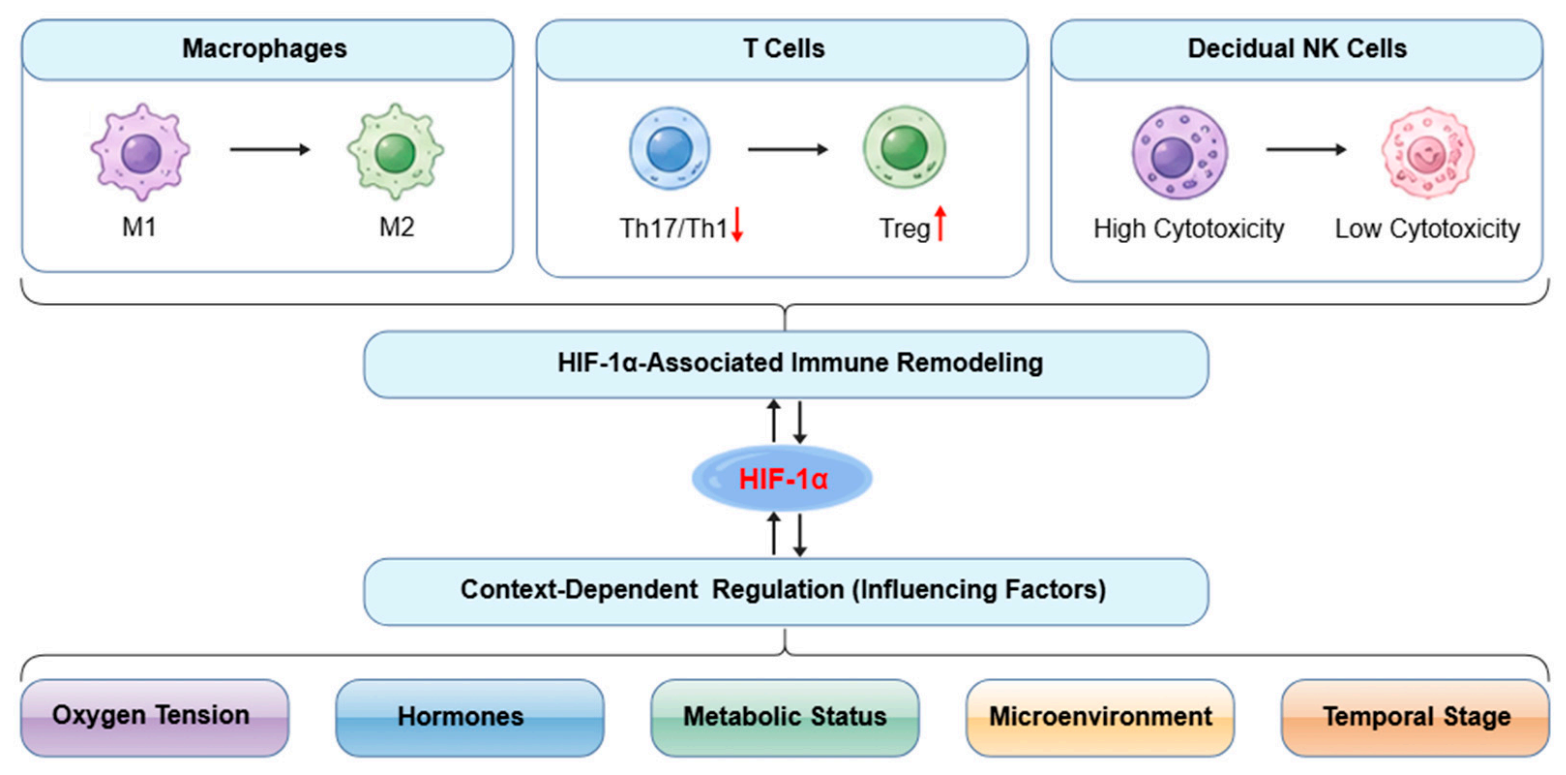
HIF-1α-associated immune remodeling. HIF signaling may influence macrophage, T-cell, and decidual NK-cell programs, but the direction and magnitude of these effects are context-dependent. The schematic therefore summarizes potential regulatory domains rather than a fixed HIF-1α-dependent immune pathway. Created with BioGDP.com (accessed on 7 August 2026).

The establishment of a normal pregnancy relies on coordinated immune regulation at the maternal–fetal interface. Immune cells, including Tregs, macrophages, dNK cells, and dendritic cells, form a substantial proportion of decidual tissue [18,19,20]. HIF signaling has been implicated in the function of several immune-cell populations in different experimental settings (Figure 9), but its specific contribution to immune remodeling in human decidual tissue remains incompletely resolved.

Macrophages contribute to tissue repair and immune regulation at the maternal–fetal interface, although their phenotypes are more diverse than a strict M1/M2 classification implies [78,79,80,81]. HIF-1α has been associated with changes in IL-10, ARG1, VEGF, and macrophage activation in selected models [21,22,23,24,25]. Whether these effects are directly mediated by HIF-1α in decidual macrophages, and how they affect debris clearance or tissue homeostasis in vivo, require further cell-specific investigation.

HIF-1α effects on T-cell differentiation are context-dependent and may vary with metabolic state, cytokine milieu, and activation conditions. Some studies have linked HIF signaling to Foxp3 expression, Treg abundance, or Th1-associated cytokine responses [82,83,84,85], but these observations do not establish a uniformly immunosuppressive action in decidual tissue. Reports of lower HIF-1α and Treg levels alongside increased Th17 cells in recurrent miscarriage are clinically associative and cannot determine causal direction [26,27]. HIF-1α may be one component of the regulatory environment, rather than a singular determinant of maternal–fetal immune balance.

Furthermore, HIF-1α is involved in regulating the functions of dNK cells. Under normal conditions, dNK cells exhibit low cytotoxicity and high pro-angiogenic activity, secreting factors such as VEGF, ANGPT and GM-CSF to promote vascular remodeling [86,87,88]. HIF-1α suppresses the expression of molecules associated with cytotoxicity and promotes the development of a tolerogenic phenotype, preventing an immune attack on the embryo [89,90].

## 6. Context-Dependent HIF-1α Regulatory Network in Decidualization and Early Pregnancy

**Figure 10:**
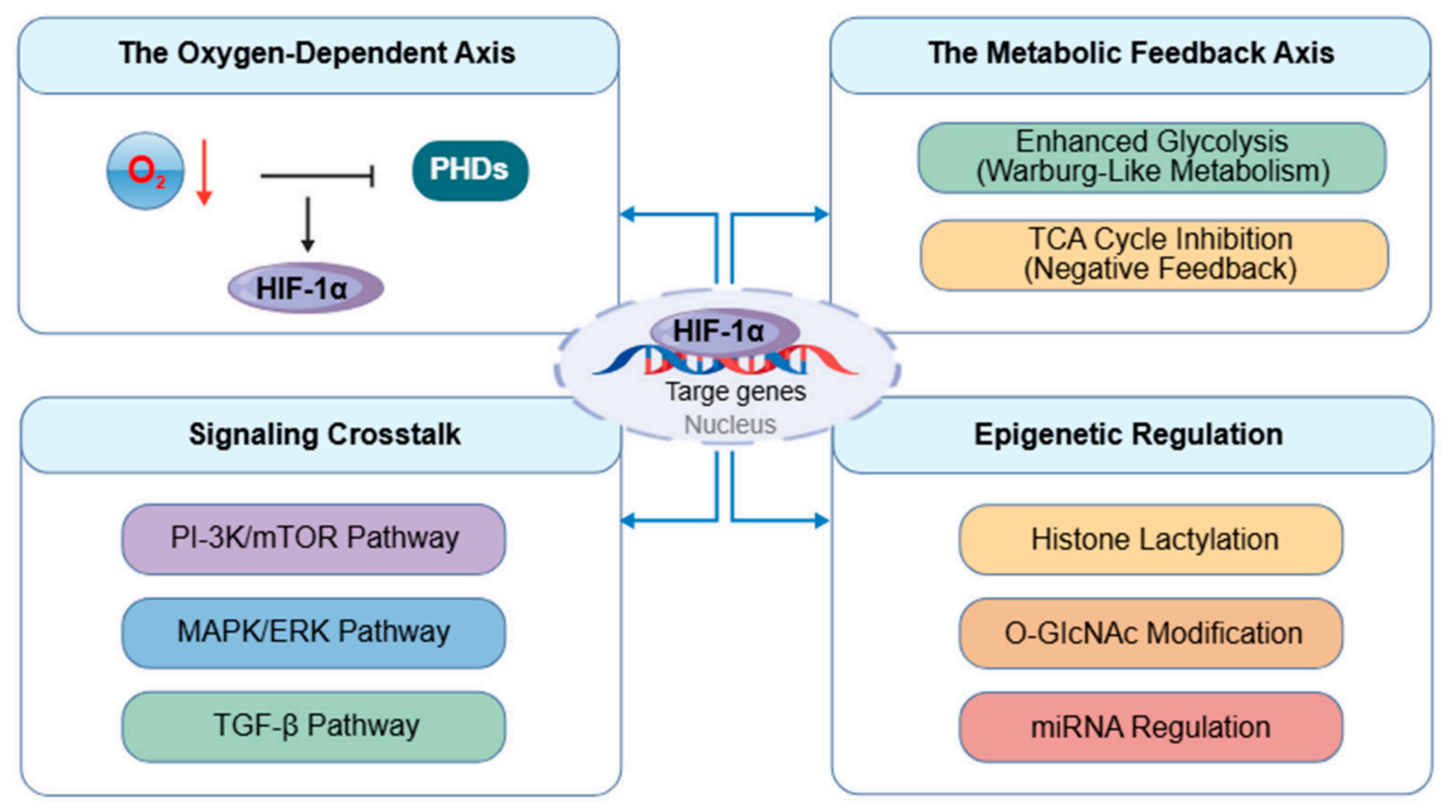
Integrated HIF-1α regulatory network during endometrial decidualization. Four major regulatory domains, oxygen sensing, metabolism, intracellular signaling, and epigenetic/post-transcriptional regulation, converge on HIF-1α-associated cellular responses. Created with BioGDP.com (accessed on 7 August 2026).

During endometrial decidualization, HIF-1α-associated signaling intersects with pathways involved in cellular differentiation, metabolism, vascular adaptation, and immune regulation [2,12]. These interactions can be considered within a multi-layered regulatory framework comprising oxygen sensing, metabolic signals, intracellular signaling, and epigenetic or post-transcriptional regulation (Figure 10). The framework is intended to organize candidate interactions; it should not be interpreted as demonstrating that HIF-1α causally coordinates all of these processes in human endometrial tissue.

### 6.1. The Oxygen-Dependent Axis: The Classical PHD/VHL Degradation System

The canonical oxygen-sensing mechanism has been described in detail in Section 4; this subsection therefore focuses on how that pathway is integrated into decidualization rather than repeating the PHD/VHL cascade. During physiological hypoxia, reduced PHD activity permits HIF-1α stabilization and transcriptional activity, whereas restoration of oxygen availability favors hydroxylation and VHL-dependent degradation. Endometrial studies indicate that components of this oxygen-sensing system vary across the menstrual cycle and implantation window, supporting a dynamic rather than constitutively activated HIF program. Importantly, changes in HIF-1α abundance should be interpreted together with oxygen tension, progesterone/cAMP signaling, and cellular metabolic state rather than as an isolated molecular event.

### 6.2. The Metabolic Feedback Axis: Lactate-α-KG Regulation of HIF Stability

Decidualization is accompanied by substantial metabolic remodeling, including changes in glycolytic activity, mitochondrial function, lipid metabolism, and TCA-cycle intermediates. HIF-1α provides a plausible oxygen-sensitive link between these metabolic changes and transcriptional adaptation, but the extent to which HIF-1α is required for the full decidual metabolic transition remains incompletely defined (Figure 10).

HIF-1α can enhance expression of glycolytic genes such as GLUT1, HK2, and LDHA, thereby favoring glycolytic flux and lactate production. Lactate and related metabolic signals may in turn influence oxygen-sensing enzymes and HIF stability in selected cellular contexts. However, the complete HIF-1α-glycolysis-lactate feedback circuit has not been established as a single causal pathway in human decidualizing stromal cells. Direct measurements of metabolic flux, lactate turnover, HIF activity, and rescue after pathway perturbation are required to determine its quantitative contribution.

In contrast to lactate, α-KG exerts a negative feedback effect on HIF-1α regulation. As a crucial cofactor for PHD-catalyzed reactions, elevated levels of α-KG promote the hydroxylation and degradation of HIF-1α. However, under conditions of hypoxia and high glycolytic activity, reduced TCA activity leads to decreased α-KG production, further promoting HIF-1α stabilization [91].

Furthermore, metabolic intermediates such as succinate and fumarate can inhibit α-KG-dependent dioxygenases and thereby influence HIF stability in specific metabolic contexts [92,93,94,95,96]. This provides a mechanistic basis for reciprocal interactions between metabolism and HIF signaling, but the quantitative contribution of these metabolites during human endometrial decidualization remains to be established.

### 6.3. Signaling Crosstalk: PI-3K/AKT/mTOR, MAPK and TGF-β Networks

In addition to classical hypoxia-dependent regulation, HIF-1α can be influenced by intracellular signaling pathways. The PI-3K/AKT/mTOR pathway is a recognized non-canonical regulator of HIF protein synthesis and activity [50,51,52]. During decidualization, progesterone and cAMP signaling also modulate intracellular pathways that can intersect with HIF-1α regulation. However, evidence for a direct progesterone-mTOR-HIF-1α sequence in human decidualizing stromal cells remains limited, and this mechanism should therefore be considered context-dependent rather than universal.

The MAPK/ERK pathway is also involved in the regulation of HIF-1α. Studies have shown that ERK activation enhances HIF-1α transcriptional activity and promotes its nuclear stability, upregulating the expression of VEGF and glycolysis-related genes [83,84,85]. Furthermore, inflammatory cytokine-induced activation of p38 MAPK enhances HIF-1α-mediated immunomodulation, highlighting its significance in the establishment of maternal–fetal immune tolerance.

Crosstalk exists between TGF-β and HIF-1α, primarily involving the regulation of extracellular matrix remodeling and fibrosis. TGF-β enhances HIF-1α transcriptional activity, while HIF-1α promotes the expression of TGF-β-associated fibrosis genes [6]. During decidualization, this synergistic interaction facilitates stromal cell remodeling and the stabilization of spiral arteries, but excessive activation may lead to aberrant fibrosis and pathological pregnancy outcomes.

### 6.4. Epigenetic Regulation: Histone Lactylation, O-GlcNAc Modification and miRNAs

Epigenetic regulation is a potentially important interface between metabolic state and HIF signaling, but the strength of evidence varies across biological systems (Figure 10). In particular, the proposed HIF-1α-lactate-histone lactylation pathway should currently be regarded as a candidate mechanism rather than an established causal circuit in decidualizing endometrial stromal cells.

Lactate can influence chromatin state through histone lactylation, and decidualization-associated metabolic changes provide a plausible biochemical context for this process. However, direct demonstration that HIF-1α-driven lactate production causes locus-specific histone lactylation and thereby controls decidual genes such as PRL or IGFBP1 remains limited. Establishing this mechanism will require temporal coupling of HIF-1α activity, metabolic flux, lactate concentration, locus-specific lactylation, gene expression, and perturbation/rescue experiments in decidualizing cells.

O-GlcNAc glycosylation also plays a role in regulating HIF-1α stability. Research has shown that O-GlcNAc transferase can directly modify the HIF-1α protein, reducing its ubiquitin-mediated degradation and enhancing its stability within the nucleus [97,98,99]. Furthermore, various miRNAs such as miR-210, miR-21 and miR-199a, have been confirmed to regulate HIF-1α expression or the activity of its downstream target genes [100,101,102]. Among these, miR-210 is a classic hypoxia miRNA, which is induced by HIF-1α under hypoxic conditions and subsequently enhances the cell’s adaptive response to hypoxia, creating a positive feedback regulatory loop [103].

## 7. Context-Dependent HIF-1α Dysregulation in Pregnancy-Related Disorders

**Figure 11:**
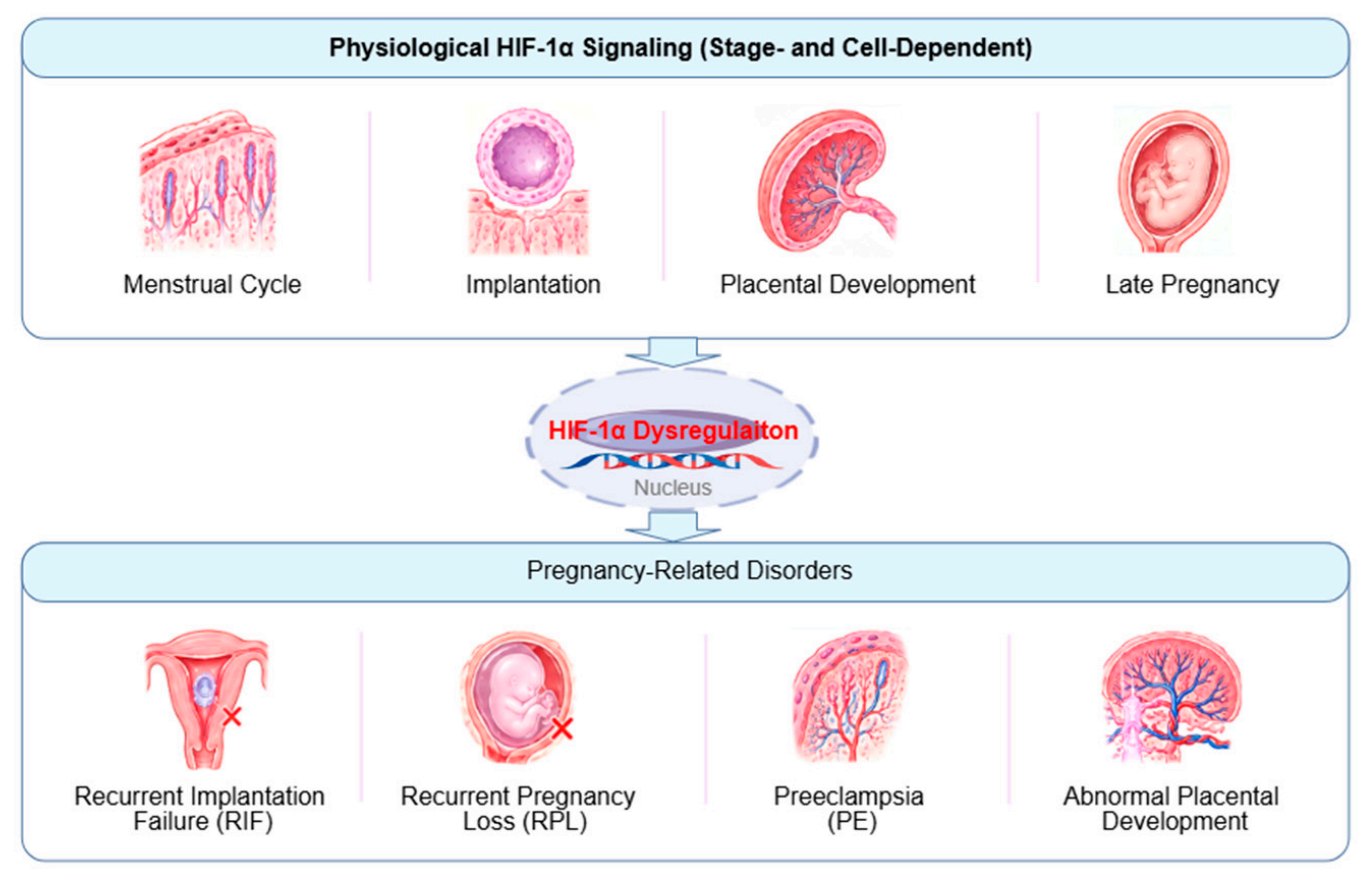
HIF-1α dysregulation in pregnancy-related disorders. Physiological HIF signaling is stage- and cell-dependent. Abnormal HIF-1α activity has been associated with recurrent implantation failure, recurrent pregnancy loss, preeclampsia, and abnormal placental development, but the underlying mechanisms differ among disorders and tissue compartments. Created with BioGDP.com (accessed on 7 August 2026).

HIF-1α abnormalities are closely associated with various pregnancy-related disorders, including RIF, recurrent pregnancy loss, preeclampsia and abnormal placental development (Figure 11) [12]. Although these conditions present with different clinical manifestations, they all involve reduced endometrial receptivity, impaired decidualization, abnormal vascular remodeling, and maternal–fetal immune imbalance. HIF-1α dysfunction is considered a common and significant pathological basis underlying these disorders [28].

### 7.1. Insufficient HIF-1α Activity in Endometrial Dysfunction

**Figure 12:**
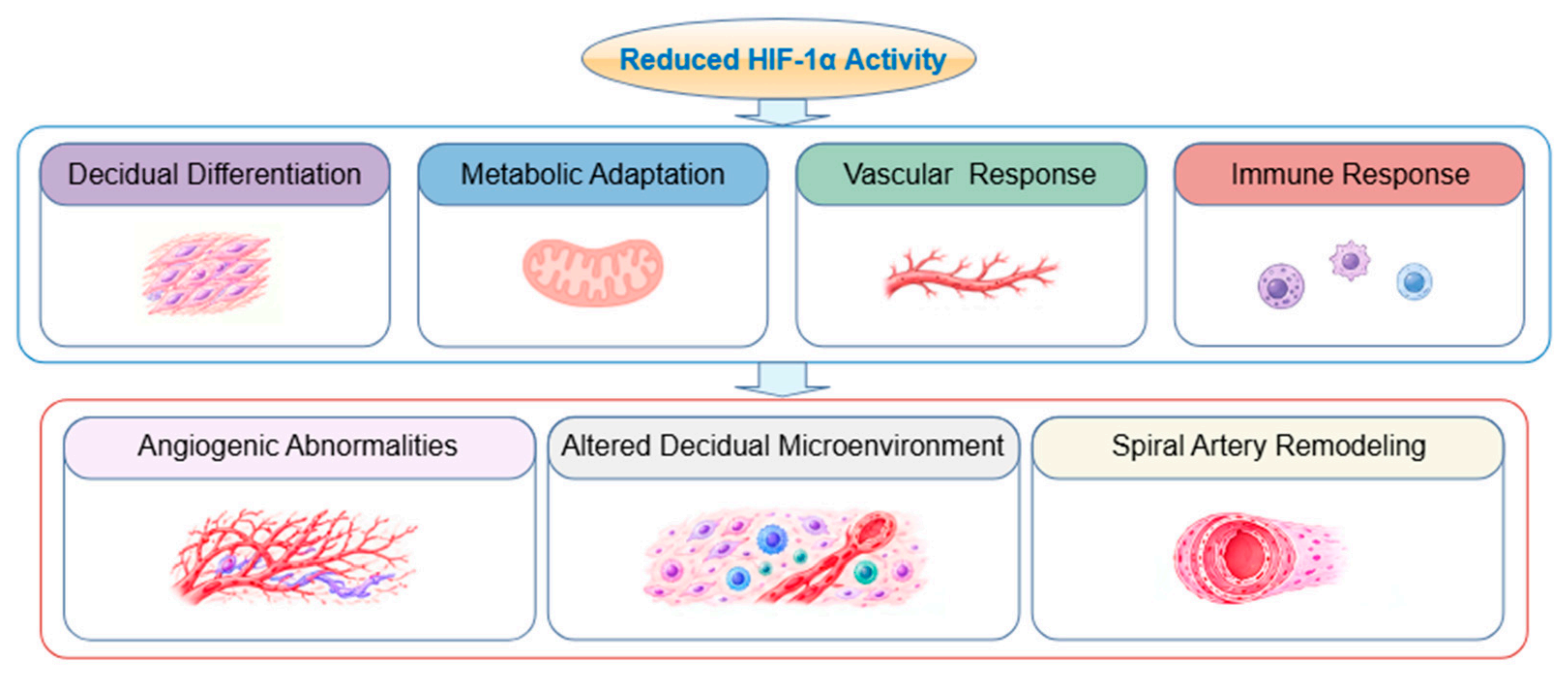
Potential consequences of insufficient HIF-1α function. Reduced HIF-1α activity may affect decidual differentiation, metabolic adaptation, and vascular or immune responses. Angiogenic abnormalities can contribute to an altered decidual microenvironment, but spiral artery remodeling is mediated by additional cell populations and should not be represented as a direct downstream consequence of impaired angiogenesis. Created with BioGDP.com (accessed on 7 August 2026).

During the peri-implantation period, HIF-1α-responsive signaling is associated with physiological endometrial adaptation and decidualization. Experimental studies suggest that reduced HIF-1α activity can alter expression of genes involved in metabolism, vascular signaling, and stromal-cell differentiation (Figure 12) [38,39,40,41,42,43]. Nevertheless, reduced HIF-1α activity may represent a cause, consequence, or marker of impaired decidualization depending on the experimental or clinical context; the available evidence does not establish HIF-1α deficiency as the sole determinant of endometrial dysfunction.

When HIF-1α expression is insufficient or its activity is reduced, the endometrium fails to establish the necessary adaptive response to hypoxia. Liang et al. have demonstrated that HIF-1α expression in the endometrium of patients with RIF is significantly lower than in women with normal pregnancies. This reduction is accompanied by decreased expression of key glycolytic enzymes, including PKM2, GLUT1, and LDHA [13]. Impaired glycolysis leads to insufficient ATP production in decidual cells, preventing them from completing the normal differentiation process, which ultimately results in reduced endometrial receptivity and embryo implantation failure.

Insufficient HIF-1α signaling may reduce angiogenic responses in the endometrium, particularly through altered VEGF-related signaling [3,4,5,10,11]. However, this should be distinguished from spiral artery remodeling, which is mediated by multiple maternal and fetal/interface cell populations, including dNK/uNK cells and EVTs. Clinical observations of reduced HIF-1α/VEGF expression occurring together with impaired vascular remodeling do not establish a direct causal sequence.

Disruption of immune regulation may accompany impaired decidualization when HIF-1α signaling is altered. Studies from maternal–fetal interface and immune-cell models suggest that HIF-1α can influence macrophage, T-cell, and dNK-cell phenotypes, but the direction and magnitude of these effects are context-dependent [18,19,20].

### 7.2. Sustained or Excessive HIF-1α Activation: A Distinct Pathological Context

**Figure 13:**
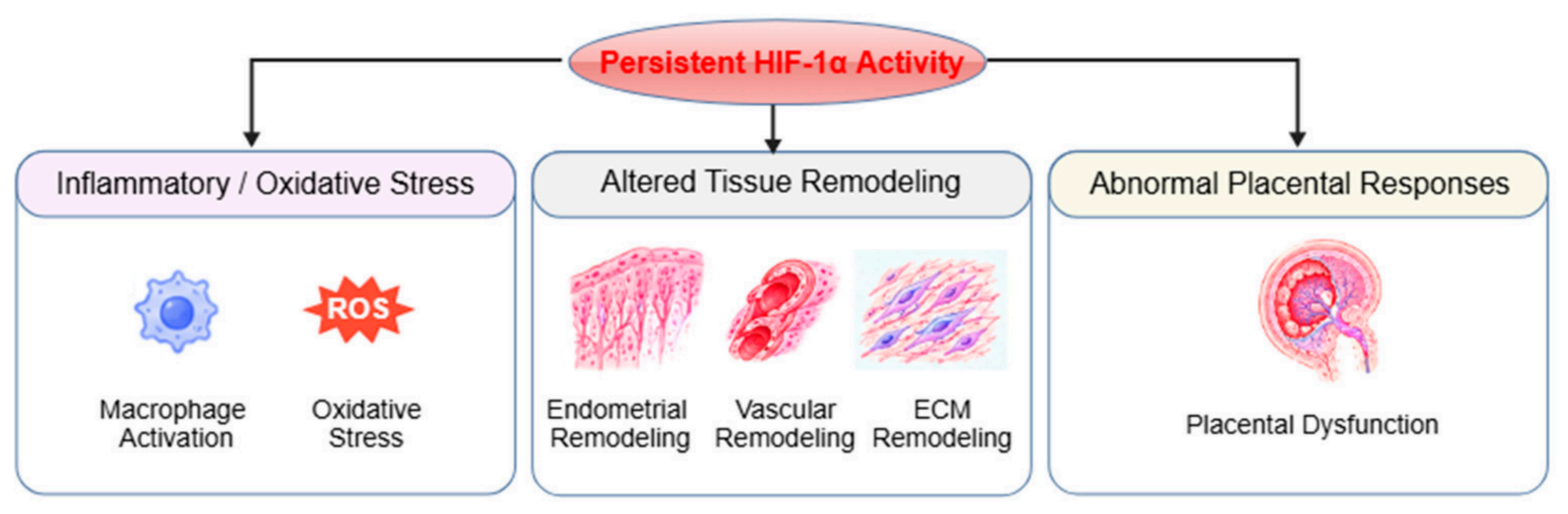
Potential consequences of persistent HIF-1α activation. Sustained HIF-1α activity may be associated with inflammatory/oxidative stress, altered tissue remodeling, and abnormal placental responses in specific pathological contexts. The outcome depends on cell type, oxygen tension, exposure duration, and gestational stage. Created with BioGDP.com (accessed on 7 August 2026).

Persistent or excessive HIF-1α activity has been associated with several pregnancy-related pathologies, including preeclampsia, in which placental hypoperfusion and pathological hypoxia can coexist with elevated HIF signaling (Figure 13) [76,77]. However, the available evidence does not establish sustained HIF-1α activation as a universal upstream cause. HIF-1α may be a component or amplifier of the pathological response, and its effects depend on the affected cell type, oxygen environment, duration of activation, and gestational stage. Thus, observations in placental or trophoblast compartments should not be directly equated with mechanisms operating in decidual stromal cells.

HIF-1α overactivation also leads to abnormal tissue remodeling. Prolonged high levels of HIF-1α can continuously activate TGF-β and MMP-related pathways, resulting in abnormal extracellular matrix deposition and an increased tendency toward fibrosis [9]. This aberrant tissue remodeling not only compromises endometrial elasticity but also affects the depth of trophoblast invasion, exacerbating placental developmental disorders. Thus, HIF-1α exerts a classic double-edged sword effect during pregnancy. Insufficient expression leads to failed decidualization, whereas sustained overactivation can trigger pathological inflammation and placental dysfunction.

## 8. Potential Therapeutic Interventions and Clinical Perspectives

The therapeutic modulation of HIF signaling is an emerging research area, but current evidence does not support routine HIF-directed treatment for decidualization disorders or pregnancy complications. Potential strategies include PHD inhibition or HIF stabilization, direct modulation of HIF transcriptional activity, and RNA-based approaches targeting HIF-regulatory networks. PHD inhibitors can stabilize HIF proteins by reducing prolyl hydroxylation, whereas HIF-directed compounds or microRNA-based approaches may alter downstream hypoxia-responsive programs [9,52,100,101,102,103]. However, these approaches have major translational limitations because physiological HIF signaling contributes to normal endometrial adaptation, vascular responses, and immune homeostasis. Systemic HIF activation may therefore produce effects in tissues other than the endometrium, and pharmacological PHD inhibition may have HIF-independent or isoform-specific actions. In addition, therapeutic effects are likely to depend on dose, exposure duration, tissue compartment, menstrual or gestational stage, and the relative contribution of HIF-1α versus HIF-2α. Future studies should prioritize localized delivery, cell-specific targeting, temporal intervention, and reproductive-safety assessment before clinical translation can be considered.

A major challenge is to distinguish restoration of a deficient physiological HIF response from indiscriminate pathway activation. Candidate interventions should therefore be evaluated using cell-specific gain- and loss-of-function models, defined oxygen conditions, and clinically relevant time windows. End points should include decidual markers, vascular adaptation, immune-cell function, metabolic flux, implantation competence, and pregnancy outcomes. These studies are particularly important because a treatment that improves one cellular compartment could disturb another. At present, HIF-targeted approaches should consequently be regarded as experimental hypotheses rather than established clinical therapies.

Several limitations should temper mechanistic interpretation. Direct causal studies in human endometrial stromal/decidual cells remain fewer than studies performed in trophoblasts, placental tissues, immune cells, or non-reproductive systems. Many clinical studies are observational and cannot determine whether altered HIF-1α activity is a cause, consequence, or biomarker of reproductive dysfunction. In addition, HIF responses are strongly dependent on oxygen level, duration of exposure, cell identity, menstrual or gestational stage, and the balance between HIF-1α and HIF-2α. Pharmacological PHD inhibitors may also exert HIF-independent or tissue-specific effects. These limitations support a cautious interpretation of proposed HIF-centered mechanisms and argue against assuming that pathway modulation will produce uniform reproductive effects.

## 9. Conclusions and Future Perspectives

Current evidence supports a role for HIF-1α in the adaptive response of the endometrium to changing oxygen and metabolic conditions, but the magnitude and direction of its effects are context-dependent. Direct evidence is strongest for associations with endometrial stromal-cell differentiation, metabolic adaptation, and endometrial receptivity, whereas several proposed immune, vascular, and epigenetic mechanisms remain incompletely validated in decidualizing human stromal cells.

An important unresolved issue is how HIF-1α and HIF-2α divide or share functions across the menstrual cycle, decidualization, implantation, and the maternal–fetal interface. Future studies should combine single-cell and spatial transcriptomics with chromatin profiling, proteomics, metabolomics, and metabolic-flux analysis to resolve cell-specific HIF networks and their temporal dynamics. These approaches should be integrated with perturbation and rescue experiments rather than relying solely on correlative multi-omics signatures.

Translationally, the central question is not whether HIF signaling can be activated, but whether its activity can be precisely modulated in the correct cell type and time window without disrupting physiological reproductive functions. Localized delivery, isoform-selective modulation, temporal control, and rigorous reproductive-safety assessment will be essential before HIF-directed interventions can be considered clinically. This context-dependent framework provides a more cautious basis for understanding HIF biology in decidualization and for designing future mechanistic and therapeutic studies.

## Data Availability

No new data were created or analyzed in this study. Data sharing is not applicable to this article.

## Abbreviations

The following abbreviations are used in this manuscript:

α-KG	α-Ketoglutarate
ANGPT	Angiopoietin
AKT	Serine/Threonine Kinase, Protein Kinase B, PKB
ARG1	Arginase-1
cAMP	Cyclic Adenosine Monophosphate
C/EBPβ	CCAAT/Enhancer-Binding Protein β
dNK	Decidual Natural Killer Cell
DSC	Decidual Stromal Cell
ERK	Extracellular Signal-Regulated Kinase
ESC	Endometrial Stromal Cell
FoxO1	Forkhead Box O1
Foxp3	Forkhead Box P3
GLUT1	Glucose transporter 1
GM-CSF	Granulocyte-Macrophage Colony-Stimulating Factor
HIF	Hypoxia-Inducible Factor
HK2	Hexokinase 2
HRE	Hypoxia Response Element
IGFBP1	Insulin-Like Growth Factor-Binding Protein 1
IL-10	Interleukin-10
IFN-γ	Interferon-γ
LDHA	Lactate Dehydrogenase A
MAPK	Mitogen-Activated Protein Kinase
MMP	Matrix Metalloproteinase
mTOR	Mammalian Target of Rapamycin
O-GlcNAc	O-linked β-N-Acetylglucosamine
PFKFB3	Phosphofructokinase
PHD	Prolyl Hydroxylase
PI-3K	Phosphatidylinositol 3-kinase
PKM2	Pyruvate Kinase M2
PR	Progesterone Receptor
PRL	Prolactin
RIF	Recurrent Implantation Failure
ROS	Reactive Oxygen Species
STAT3	Signal Transducer and Activator of Transcription 3
TCA	Tricarboxylic Acid Cycle
TGF-β	Transforming Growth Factor-β
Th1	T Helper Type 1 Cell
Th17	T helper Type 17 Cell
TNF-α	Tumor Necrosis Factor-α
Tregs	Regulatory T Cell
VEGF	Vascular Endothelial Growth Factor
VHL	Von Hippel–Lindau Tumor Suppressor
